# Partially hydrolyzed guar gum increased colonic mucus layer in mice via succinate-mediated MUC2 production

**DOI:** 10.1038/s41538-023-00184-4

**Published:** 2023-03-28

**Authors:** Mariko Kajiwara-Kubota, Kazuhiko Uchiyama, Kohei Asaeda, Reo Kobayashi, Hikaru Hashimoto, Takeshi Yasuda, Satoshi Sugino, Takeshi Sugaya, Yasuko Hirai, Katsura Mizushima, Toshifumi Doi, Ken Inoue, Osamu Dohi, Naohisa Yoshida, Takeshi Ishikawa, Tomohisa Takagi, Hideyuki Konishi, Ryo Inoue, Yoshito Itoh, Yuji Naito

**Affiliations:** 1 Department of Molecular Gastroenterology and Hepatology, Kyoto Prefectural University of Medicine, Kyoto, 602-8566 Japan; 2 Department of Medical Regulatory Science, Kyoto Prefectural University of Medicine, Kyoto, 602-8566 Japan; 3 Department of Human Immunology and Nutrition Science, Kyoto Prefectural University of Medicine, Kyoto, 602-8566 Japan; 4 Department for Medical Innovation and Translational Medical Science, Graduate School of Medical Science, Kyoto Prefectural University of Medicine, Kyoto, 602-8566 Japan; 5 Laboratory of Animal Science, Department of Applied Biological Sciences, Faculty of Agriculture, Setsunan University, Hirakata, 572-8508 Japan

**Keywords:** Gastrointestinal system, Nutrition

## Abstract

Colonic mucus layers protect intestinal tissues against intestinal bacteria. We investigated the effects of dietary fiber and its metabolites on mucus production in the colonic mucosa. Mice were fed a partially hydrolyzed guar gum (PHGG)-containing diet and a fiber-free diet (FFD). The colon mucus layer, fecal short-chain fatty acid (SCFA) levels, and gut microbiota were evaluated. Mucin 2 (MUC2) expression was assessed in SCFA-treated LS174T cells. The role of AKT in MUC2 production was investigated. The mucus layer in the colonic epithelium was significantly increased in the PHGG group compared with that in the FFD group. In the PHGG group, an increase in Bacteroidetes in the stool was observed, and fecal acetate, butyrate, propionate, and succinate levels were significantly increased. However, MUC2 production was significantly increased only in succinate-stimulated LS174T cells. The succinate-induced MUC2 production was associated with AKT phosphorylation. Succinate mediated the PHGG-induced increase in the colon mucus layer.

## Introduction

The maintenance of the intestinal epithelium in the colon is known to involve processes, such as mucus production, intercellular adhesion, and cytoprotection^1^. The colonic mucus layer consists of ~30 core proteins including mucins and antimicrobial peptides. Among these, MUC2, which is synthesized by intestinal goblet cells, is the most important component^2,3^. Goblet cells are critical for the maintenance of the colonic barrier, both through the production of mucus and transportation and presentation of luminal antigens to tolerogenic dendritic cells. Mucin, a glycoprotein produced by goblet cells, constitutes the mucus layer of the colon and plays an important role as a mucosal barrier that physically prevents the entry of luminal pathogenic bacteria into intestinal tissues^4^. It has been reported that MUC2 deficient mice develop spontaneous colitis^5^.

Dietary fiber is considered an important factor for mucus secretion in the colon and a fiber-free diet was found to markedly reduce the MUC2 mucus layer in the colon^6^. An important mechanism of mucus production by dietary fiber has been reported to involve short-chain fatty acids (SCFAs), which are dietary fiber metabolites produced by intestinal microbiota. Microbial SCFA production is essential for gut integrity as SCFAs regulate the luminal pH and mucus production, provide fuel for epithelial cells, and affect mucosal immune function^7^. Partially hydrolyzed guar gum (PHGG), which is a soluble dietary fiber produced from guar gum, has been reported to have various functions, such as the enhancement of colonic epithelial wound healing^8^, reduction of the IFNγ-induced increase in the permeability of colonic cells^9^, and anti-inflammatory effects on the suppression of dextran sulfate sodium (DSS)- and 2,4,6-trinitrobenzene sulfonic acid (TNBS)-induced colitis^10,11^. However, it is unclear whether PHGG and its metabolites are involved or not in mucus production in the colonic epithelium. In the present study, the effect of SCFAs, intestinal metabolites of PHGG, on mucus production in the colonic epithelium and the underlying mechanism were investigated.

## Results

### PHGG increased the mucus layer thickness

**Fig. 1:**
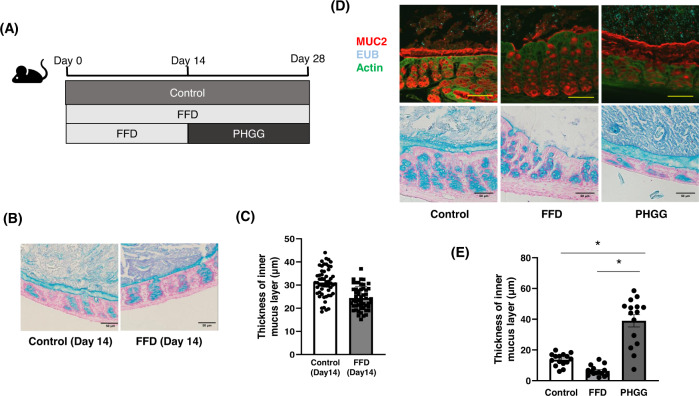
Thickness of the colon mucus layer in mice. **A** Schematic diagram of the mouse model of dietary fiber intake. **B** Thickness of the colon mucus layer in mice. Alcian blue staining of colon sections at day 14. Scale bars, 50 μm. **C** Measurement of the colonic mucus layer thickness of day 14 mice. The colonic mucus layer thickness of each group was measured at nine points, and the means were compared. **D** Fluorescent immunostaining of MUC2 in the colon of mice and alcian blue staining of sections of the colon at day 28. Scale bars, 50 μm. **E** Measurement of the colonic mucus layer thickness. The colonic mucus layer thickness of each group was measured at nine points, and the means were compared. Error bars represent the SEM. (*p < 0.01). FFD fiber-free diet, PHGG partially hydrolyzed guar gum, MUC2 mucin 2.

Mice were divided into three groups according to differences in dietary fiber intake (Fig. 1A). When mice were sacrificed at day 14, the alcian blue staining showed that the thickness of the mucus layer in the fiber-free diet (FFD)-fed group was reduced compared to that in the control group (Fig. 1B, C). The thickness of the mucus layer in the colon was compared among the three groups: control diet-fed, FFD-fed, and PHGG-fed groups (Fig. 1D). The thickness of the mucus layer was significantly decreased in the FFD-fed group compared with that in the control group. In contrast, the thickness of the mucosal layer was significantly increased in the PHGG-fed group compared with that of the control and FFD-fed groups (Fig. 1E).

### Effect of FFD and PHGG on fecal SCFA levels

**Fig. 2:**
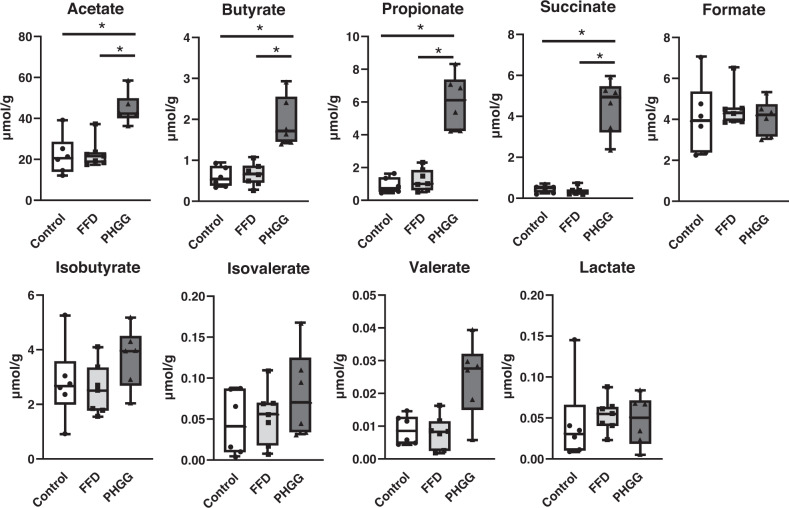
Concentration of short-chain fatty acids in fresh stool. *p < 0.05.

Among the SCFAs measured in stool in each group, PHGG administration resulted in significantly higher levels of acetate, butyrate, propionate, and succinate compared with those in the control and FFD-fed groups (Fig. 2).

### Effect of FFD and PHGG on intestinal microbiota

**Fig. 3:**
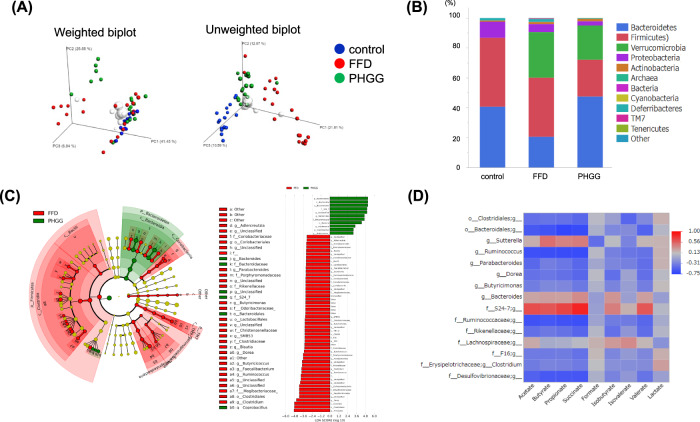
Data for analysis of intestinal bacteria in fresh mouse stool. **A** Principal component analysis. **B** Percentage of gut bacteria at the phylum level. **C** Genus-level analysis of intestinal bacteria using Lefse. **D** Heat map on the correlation between intestinal bacteria and short-chain fatty acids.

Beta diversity analysis was used to compare and analyze the microbial community structure among the three groups. Principal coordinate analysis (PCoA) showed that PHGG and FFD samples were highly segregated and that the composition of the gut microbiota differed significantly between them (Fig. 3A). The abundance of Bacteroidetes and Firmicutes was high in the control, FFD, and PHGG groups. Furthermore, in the PHGG-fed group, the abundance of Firmicutes decreased and that of Bacteroidetes increased compared to that in the FFD-fed group (Fig. 3B, C). Analysis at the phylum level showed that the percentages of Bacteroides and S24-7 significantly increased in the PHGG group. Bacterial species with significant differences between the FFD and PHGG groups were selected, and the correlation between these bacteria and SCFA levels was analyzed. Among the SCFAs, acetate, butyrate, propionate, and succinate levels were elevated in the PHGG group, and were correlated with the abundance of Sutterella, S24-7, and Bacteroides. In particular, succinate was strongly positively correlated with S24-7 abundance (Fig. 3D).

### Mucus layer thickness in the stool transplant model

**Fig. 4:**
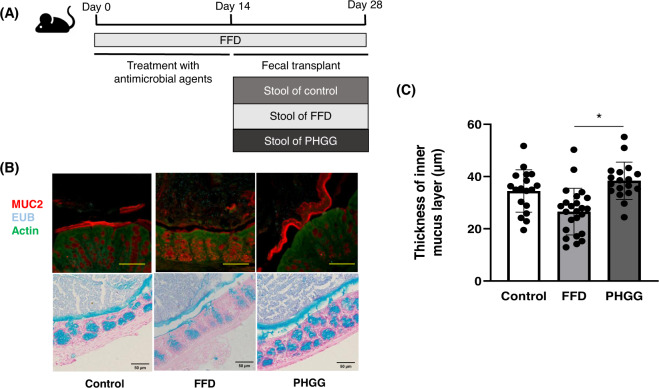
Thickness of the colon mucus layer in fecal transplant model mice. **A** Schematic diagram of the stool transplant mouse model. **B** Fluorescent immunostaining of MUC2 in the colon of mice and alcian blue staining of sections of the colon. Scale bars, 50 μm. **C** Measurement of the colonic mucus layer thickness. In each group, the colonic mucus layer thickness was measured at nine points and the means were compared. Error bars represent the SEM. (*p < 0.05).

The stool transplant models, in which stool of the control diet-fed, FFD-fed, and PHGG-fed groups was used, are shown in Fig. 4A. As shown in Fig. 4B, C, the thickness of the mucus layer was significantly increased in the PHGG stool transplant group and decreased in the FFD stool transplant group compared to that in the control group.

### MUC2 production by mucus-producing cells

**Fig. 5:**
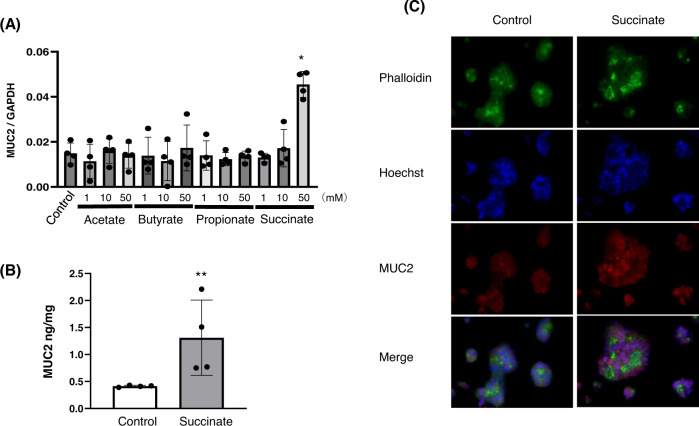
Mucin 2 (MUC2) production in colon mucus cells, LS174T cells. **A** MUC2 mRNA expression in control cells and LS174T cells treated with four short-chain fatty acids (acetate, butyrate, propionate, succinate) (*p < 0.01, vs. all). **B** ELISA for MUC2 in control and succinate-treated cells (**p < 0.05, vs. control). **C** Fluorescence imaging of control, LS174T cells, and succinate-treated cells stained with rhodamine-phalloidin and Hoechst.

To confirm the induction of MUC2 by SCFAs, LS174T cells were treated with SCFAs and MUC2 mRNA expression was examined. Four types of SCFAs (acetate, butyrate, propionate, and succinate), whose levels were increased in the stool following PHGG administration to mice, were administered. The MUC2 mRNA and protein levels in LS174T cells significantly increased only after succinate administration (Fig. 5A, B, respectively). Fluorescence immunostaining also showed MUC2 induction by succinate in LS174T cells (Fig. 5C).

### Succinate-induced MUC2 production was mediated by AKT phosphorylation

**Fig. 6:**
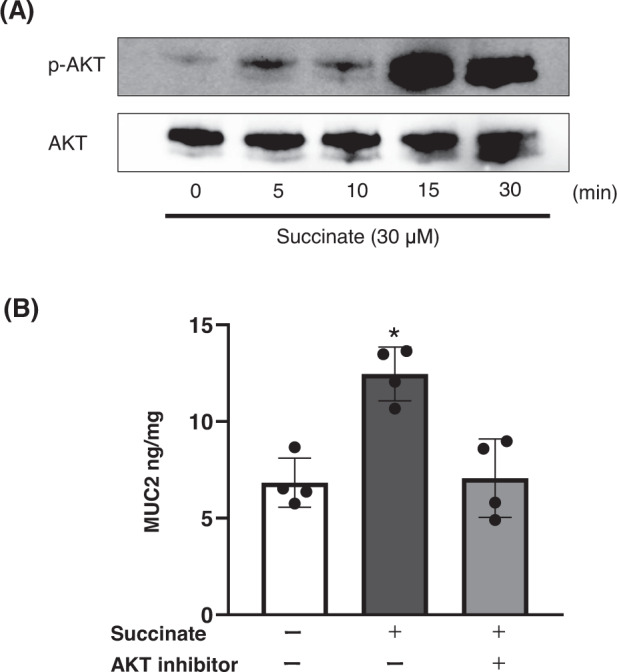
AKT pathway in colon mucus production. **A** MUC2 protein expression on the control and succinate-transfected LS174T cells. **B** MUC2 protein in LS174T cells pretreated with 10 μM of AKT inhibitor (perifosine) for 2 h and treated with succinate (*p < 0.05, vs. all).

Western blot analysis showed that AKT phosphorylation was significantly higher 30 min after succinate treatment (Fig. 6A). Pretreatment with perifosine, an AKT inhibitor, significantly prevented succinate-induced MUC2 production (Fig. 6B).

## Discussion

In the present study, we demonstrated the role of succinate in the induction of MUC2 expression in colonic epithelial cells. Succinate, one of the SCFAs metabolized from dietary fiber by the intestinal microbiota, has not been reported to play a role in MUC2 production by colonic epithelial cells. We found that intake of PHGG, which is a soluble dietary fiber, increased the mucus layer thickness on the colonic epithelium and the concentration of succinate in the stool. The increase in succinate levels with PHGG administration was corroborated by the increase in succinate-producing intestinal microbiota induced by PHGG. Furthermore, we found that succinate induces MUC2 production in mucus-producing cells via AKT phosphorylation. This study revealed that succinate may mediate the dietary fiber intake-induced increase in the colonic mucus layer, resulting from the metabolism of dietary fiber by intestinal microbiota.

Dietary fiber intake stimulates SCFA production by intestinal microbiota. SCFAs (mainly acetic acid, propionic acid, and butyric acid) are important nutrients for epithelial cells^10^. SCFAs have also been reported to affect host health at the cellular, tissue, and organ levels by mechanisms related to gut barrier function, glucose homeostasis, immunomodulation, and obesity^12^. However, few reports have clarified the relationship between SCFAs and colonic epithelial mucus production. PHGG is a water-soluble dietary fiber made from guar gum, which is used as a food stabilizer and thickener^13^. PHGG intake has been reported to be effective in improving hyperlipidemia^14^ and postprandial blood sugar levels in diabetes^15^. Several studies have reported that PHGG promotes wound healing in the colonic epithelium and inhibits intestinal inflammation in DSS- and TNBS-induced colitis^10,11^. Furthermore, Takagi et al. reported that PHGG intake increased the levels of succinate, propionate, and butyrate in the stool^11^. In the present study, PHGG intake also induced specific SCFAs, similar to previous reports on stool.

Recently, symbiosis between intestinal bacteria and the host has been shown to be beneficial for both the host and bacteria, which are considered to have a symbiotic relationship. SCFAs are considered the major metabolites, both quantitatively and qualitatively, by which gut bacteria produce beneficial effects on the host^16^. Succinate is an important metabolite in both host and microbial processes. Numerous studies support that succinate is not simply an inert byproduct of metabolism but that it also plays an active role in downstream cellular responses and that it can have tissue-specific and systemic effects as a pro-inflammatory mediator^17–20^. It has been reported that succinate induces inflammation in the colon via SUCNR1^21^. Furthermore, succinate levels were found to be elevated and SUCNR1 expression to be increased in tissues from patients with inflammatory bowel disease. Moreover, succinate levels promoted phosphorylation of the pro-fibrotic transcription factors STAT3 and NF-kB via SUCNR1, which induces inflammation^21^. However, it has also been reported that succinate accumulation improves glucose metabolism^22^. A diet containing fiber elevates the levels of succinate in the cecum as a substrate for intestinal glycogenesis, which improves glucose homeostasis. De Vadder also reported that dietary succinate improves glucose and insulin tolerance^22^. Hakak et al. reported that succinate promotes hematopoietic cell proliferation via phosphorylation and activation of the MAPK pathway^23^. Thus, it has been revealed that succinate is not merely an intermediate product of metabolism, but that it has various effects on the body. In the present study, we observed a marked increase in the levels of four SCFAs in the PHGG group, of which succinate was the most representative. Succinate-producing intestinal bacteria belong to the phylum Bacteroides, which was observed in the PHGG group in this study.

Strain S24-7, which belongs to the phylum Bacteroides, is an unclassified anaerobic bacterium^24^. PHGG administration has been reported to increase the abundance of S24-7 in the colon of a mouse model. Furthermore, the abundance of S24-7 was found to be increased in mice fed a low-fat diet, producing abundant SCFAs^24^. In a mouse colitis model, intestinal stress induced strains of bacteria, such as S24-7, and these bacteria suppressed inflammation in the intestine^25^. Although not all of the actions of S24-7 have been elucidated, the association between SCFA production and S24-7 is unclear. In the present study, PHGG intake also increased the number of S24-7 strains in the colon and the levels of SCFAs, such as propionate, butyrate, acetate, and succinate. Among these SCFAs, a strong correlation between S24-7 and succinate levels was demonstrated. These results indicate that the administration of PHGG increases the levels of succinate in the intestine via an increase in the abundance of S24-7.

The mucin protein family consists of MUC1, MUC2, MUC3, MUC4, MUC5A, and MUC5AC. MUC2 is the major mucin on the colon surface^26^. The PI3-Akt pathway is an intracellular signaling pathway involved in protein synthesis and proliferation. Mucus-producing cells in the lung have been reported to produce MUC5A via the ERK and AKT pathways^27^. In addition, the association between MUC5AC and the AKT pathway has been reported in nasal epithelial cells^28^. Based on these results, mucus production might be associated with the AKT pathway, but there are few reports showing an association between the PI3-AKT pathway and mucus production in the intestinal epithelium. In the present study, succinate-induced AKT phosphorylation in mucus-producing cells and MUC2 production induced by succinate were inhibited by AKT inhibitors, suggesting that the AKT pathway is associated with MUC2 in the colon.

In conclusion, we report the succinate-induced production of MUC2 in the colonic epithelium for the first time. MUC2 production in the colonic epithelium is important for mucin formation in the colon, and succinate may play an important role in protecting the colonic epithelium by constructing a mucosal barrier.

## Methods

### Mouse model of dietary fiber

Six-week-old C57BL6 mice were divided into the following three groups: control diet (AIN93G combined 5% cellulose)-fed, FFD-fed, and PHGG-blended diet-fed (PHGG-fed) groups. The FFD consisted of AIN93G plus cornstarch instead of cellulose, and the PHGG-blended diet consisted of AIN93G plus PHGG instead of cellulose. These three mouse groups were maintained for 4 weeks, and the MUC2 mucus layer in the colon and SCFA in the stool were measured.

### Fecal transplant mouse model

Fecal samples were collected from the mice fed the control, FFD, and PHGG diets, as well as from the model mice of Fig. 1A. The collected stool was suspended in PBS, centrifuged, and the supernatant was collected. Six-week-old mice were treated with three antimicrobial agents (vancomycin 0.5 g/l, ampicillin 1 g/l, neomycin1 g/l) for 2 weeks, and then forced to receive 0.2 ml of stool supernatant every other day for 2 weeks.

### Analysis of the mucus layer thickness in the colon

Collected colon tissues were fixed in Karnois solution for 3 h and then immersed in ethanol. Alcian blue staining was performed on colon sections. The thickness of MUC2 was measured at nine locations in each group under ×40 magnification and the average value was used for the study.

### SCFA analysis

A portion of the cecal contents (0.3 g) was collected and suspended in 0.5 ml of 14% perchloric acid to remove proteins. After centrifugation at 10,000 × *g* for 5 min at 4 °C, the resulting supernatant was filtered through a cellulose acetate membrane filter with a pore size of 0.45 μm. The organic acid content was analyzed using ion-exclusion high-performance liquid chromatography.

### Cell culture

LS174T (CL‑188TM; American Type Culture Collection [ATCC, Manassas, VA, USA]) is a human colon adenocarcinoma cell line. LS174T exhibits the characteristics of mucin-secreting intestinal epithelial cells and is widely used as an intestinal goblet cell line. LS174T cells were cultured for 1 week in Eagle’s minimum essential medium (EMEM) supplemented with 10% heat-inactivated fetal bovine serum (FBS), 100 U/ml penicillin, and 100 U/ml streptomycin at 37 °C in a humidified incubator with 5% CO_2_ and 95% air. LS174T cells (2.5 × 10^5^ cells/ml) were seeded in 6‑well plates for the ELISA assay and 24‑well plates for the polymerase chain reaction (PCR). The LS174T cells were serum-starved for 6 h in glucose-free EMEM, prior to all cell experiments.

### Treatment of LA174T cells with SCFAs

LS174T cells were cultured in a 24-well-plate or μ-dish (35-mm) imaging dishes (Ibidi GmbH, Martinsried, Germany) until they reached 100% confluence.

Then, cells were treated with 10 μM AKT inhibitor (perifosine) for 1 h before succinate treatment. Acetate (FUJIFILM Wako Pure Chemical Co., Ltd.), butyrate (Nu-Check Prep, Inc, Waterville, MN, USA), propionate (Nu-Check Prep, Inc, Waterville, MN, USA), and succinate (Nu-Check Prep, Inc, Waterville, MN, USA) were dissolved in phosphate-buffered saline (PBS) in this experiment.

### mRNA analysis

MUC2 mRNA expression in the cells was determined using qRT-PCR. Total RNA was isolated using the acid guanidinium phenol chloroform method (TRIzol Reagent), according to the manufacturer’s instructions.

RNA concentration was determined from the absorbance at 260 nm relative to that at 280 nm. The isolated RNA samples were stored at 80 °C until use. RNA was converted into cDNA using the High Capacity cDNA Reverse Transcription Kit (Applied Biosystems). The resultant cDNA was subjected to qRT-PCR using the following primer sets: human MUC2, 5- TGGGTGTCCTCGTCTCCTACA-3, and antisense 5-TGTTGCCAAACCGGTGGTA-3; human GAPDH, sense 5-ACCACAGTCCATGCCATCACT-0, and antisense 5-CCATCACGCCACAGTTTCC-3. The Power SYBR Green PCR Master Mix and a real-time PCR system (7300; Applied Biosystems, Foster City, CA, USA) were used, and the PCR conditions included 40 cycles at 95 °C for 15 s and primer annealing at 60 °C for 1 min, with a subsequent melting curve analysis in which the temperature was increased from 60 to 95 °C. Gene expression levels were calculated relative to the levels of β-actin or GAPDH.

### ELISA assay

To measure the levels of secreted MUC2, LS 174 cells were cultured and treated with 30 mM succinate for 48 h. The concentration of MUC2 protein in the cell supernatant was measured using a Mucin ELISA kit (SEA705HU) according to the manufacturer’s instructions.

### Fluorescence microscopy

LS174T cells were seeded on μ-dish (35-mm) imaging dishes (ibidi GmbH, Martinsried, Germany) and treated with 30 mM succinate for 12 h. Then, the cells were fixed in 4% paraformaldehyde in PBS for 10 min, permeabilized with PBS containing 0.1% Triton X-100 for 5 min, and incubated with a primary antibody against MUC2 as (ab97386) for 24 h at 37°C. The cells were then incubated with rhodamine-phalloidin and Hoechst 33352 to stain the F-actin and nuclear chromatin, respectively. Thereafter, the cells were examined using an All-In-One Fluorescence Microscope BZ-X810 laser scanning microscope (Keyence, United Kingdom).

### Western blot analysis

For western blotting, cells were washed twice with ice-cold PBS and lysed with CelLytic M cell lysis agent (Sigma-Aldrich) supplemented with a protease inhibitor cocktail (Sigma-Aldrich). Lysed cells were collected using a cell scraper and centrifuged at 10,000 × *g* for 20 min at 4 °C. The supernatants were collected and protein concentrations were determined using the Bradford method. The samples were boiled at 70 °C for 10 min in LDS sample buffer, separated using 10% sodium dodecyl sulfate-polyacrylamide gel electrophoresis, and transferred onto polyvinylidene difluoride membranes (Invitrogen, Waltham, MA, USA) using a semi-dry transfer kit (Invitrogen). The membranes were treated with Tris-buffered saline (in double-distilled water) containing EzBlock (ATTO Corporation, Tokyo, Japan) at room temperature for 30 min and then incubated with primary antibodies, anti-phospho AKT (1:1000; catalog no. ser473), and anti-AKT (1:1000; catalog no. C67E7) (both from Cell Signaling Technology, Danvers, MA, USA), overnight at 4 °C. After washing with Tris-buffered saline containing 0.1% Tween 20, the membranes were incubated with anti-rabbit horseradish peroxidase IgG secondary antibodies (Santa Cruz Biotechnology) for 1 h at room temperature. Immunocomplexes were visualized using an ECL Plus Detection System (GE Healthcare, UK). Buckinghamshire, UK). Quantitative analysis was performed using the IMAGEQUANT TL software (GE Healthcare).

### Statement of ethics

The animal experiments were approved by the Institutional Animal Care and Use Committee of Kyoto Prefectural University of Medicine (Kyoto, Japan) under Assurance Number M 2021-129.

### Statistical analysis

Analysis of variance (ANOVA) was performed to assess the trend of the mean stratified according to normally distributed continuous variables. The trend test was based on a linear contrast. All analyses were performed using JMP PRO version 14.0.0 (SAS Institute Japan Ltd). Continuous data were described as the mean ± standard deviation (SD) if normally distributed, or median and interquartile range (IQR) (25%, 75%) if not normally distributed.

### Reporting summary

Further information on research design is available in the Nature Research Reporting Summary linked to this article.

## Supplementary information

Reporting Summary

## Data Availability

The datasets generated and analyzed during the current study are not publicly available due to a license agreement with Taiyo Kagaku Co., Ltd. but are available from the corresponding author on reasonable request.
