# Author Correction: Partially hydrolyzed guar gum increased colonic mucus layer in mice via succinate-mediated MUC2 production

**DOI:** 10.1038/s41538-023-00193-3

**Published:** 2023-04-22

**Authors:** Mariko Kajiwara-Kubota, Kazuhiko Uchiyama, Kohei Asaeda, Reo Kobayashi, Hikaru Hashimoto, Takeshi Yasuda, Satoshi Sugino, Takeshi Sugaya, Yasuko Hirai, Katsura Mizushima, Toshifumi Doi, Ken Inoue, Osamu Dohi, Naohisa Yoshida, Takeshi Ishikawa, Tomohisa Takagi, Hideyuki Konishi, Ryo Inoue, Yoshito Itoh, Yuji Naito

**Affiliations:** 1 Department of Molecular Gastroenterology and Hepatology, Kyoto Prefectural University of Medicine, Kyoto, 602-8566 Japan; 2 Department of Medical Regulatory Science, Kyoto Prefectural University of Medicine, Kyoto, 602-8566 Japan; 3 Department of Human Immunology and Nutrition Science, Kyoto Prefectural University of Medicine, Kyoto, 602-8566 Japan; 4 Department for Medical Innovation and Translational Medical Science, Graduate School of Medical Science, Kyoto Prefectural University of Medicine, Kyoto, 602-8566 Japan; 5 Laboratory of Animal Science, Department of Applied Biological Sciences, Faculty of Agriculture, Setsunan University, Hirakata, 572-8508 Japan

**Keywords:** Gastrointestinal system, Nutrition

Correction to: *npj Science of Food* 10.1038/s41538-023-00184-4, published online 28 March 2023

“In this article the author name Mariko Kajiwara-Kubota was incorrectly written as Mariko Kajiwara-Kubtota. The original article has been corrected.”

