# Supplementary material for: Partially hydrolyzed guar gum increased colonic mucus layer in mice via succinate-mediated MUC2 production
Source: NPJ Sci Food. 2023 Mar 28;7:10. doi: 10.1038/s41538-023-00184-4 (PMC10050209; doi:10.1038/s41538-023-00184-4)
Supplement: Supplementary file 1 — Reporting Summary [file 41538_2023_184_MOESM1_ESM.pdf]

nature portfolio

Corresponding author(s): Kazuhiko Uchiyama

Last updated by author(s): Dec 26, 2022

Reporting Summary

Nature Portfolio wishes to improve the reproducibility of the work that we publish. This form provides structure for consistency and transparency in reporting. For further information on Nature Portfolio policies, see our Editorial Policies and the Editorial Policy Checklist.

Please do not complete any field with "not applicable" or n/a. Refer to the help text for what text to use if an item is not relevant to your study.
For final submission: please carefully check your responses for accuracy; you will not be able to make changes later.

Statistics

For all statistical analyses, confirm that the following items are present in the figure legend, table legend, main text, or Methods section.

n/a	Confirmed	
☐	☒	The exact sample size (n) for each experimental group/condition, given as a discrete number and unit of measurement
☐	☒	A statement on whether measurements were taken from distinct samples or whether the same sample was measured repeatedly
☐	☒	The statistical test(s) used AND whether they are one- or two-sided<br>*Only common tests should be described solely by name; describe more complex techniques in the Methods section.*
☐	☒	A description of all covariates tested
☐	☒	A description of any assumptions or corrections, such as tests of normality and adjustment for multiple comparisons
☐	☒	A full description of the statistical parameters including central tendency (e.g. means) or other basic estimates (e.g. regression coefficient) AND variation (e.g. standard deviation) or associated estimates of uncertainty (e.g. confidence intervals)
☒	☐	For null hypothesis testing, the test statistic (e.g. F, t, r) with confidence intervals, effect sizes, degrees of freedom and P value noted<br>*Give P values as exact values whenever suitable.*
☒	☐	For Bayesian analysis, information on the choice of priors and Markov chain Monte Carlo settings
☒	☐	For hierarchical and complex designs, identification of the appropriate level for tests and full reporting of outcomes
☒	☐	Estimates of effect sizes (e.g. Cohen's d, Pearson's r), indicating how they were calculated

Our web collection on statistics for biologists contains articles on many of the points above.

Software and code

Policy information about availability of computer code

Data collection	No software was used.
Data analysis	All analyses were performed using JMP PRO version 14.0.0 318 (SAS Institute Japan Ltd).

For manuscripts utilizing custom algorithms or software that are central to the research but not yet described in published literature, software must be made available to editors and reviewers. We strongly encourage code deposition in a community repository (e.g. GitHub). See the Nature Portfolio guidelines for submitting code & software for further information.

Data

Policy information about availability of data

All manuscripts must include a data availability statement. This statement should provide the following information, where applicable:

- Accession codes, unique identifiers, or web links for publicly available datasets
- A description of any restrictions on data availability
- For clinical datasets or third party data, please ensure that the statement adheres to our policy

The datasets used and analyzed during the current study are available from the corresponding author upon reasonable request.

Human research participants

Policy information about studies involving human research participants and Sex and Gender in Research.

Reporting on sex and gender	This information has not been collected.
Population characteristics	This information has not been collected.
Recruitment	This information has not been collected.
Ethics oversight	This information has not been collected.

Note that full information on the approval of the study protocol must also be provided in the manuscript.

Field-specific reporting

Please select the one below that is the best fit for your research. If you are not sure, read the appropriate sections before making your selection.

☒ Life sciences ☐ Behavioural & social sciences ☐ Ecological, evolutionary & environmental sciences

For a reference copy of the document with all sections, see nature.com/documents/nr-reporting-summary-flat.pdf

Life sciences study design

All studies must disclose on these points even when the disclosure is negative.

Sample size	We have used 6 mice of each group for the study of Figure 1-3. We have used 6 mice of each group for the study of Figure 4 (fecal transplant model). For the cell culture experiment, all study were conducted at least 3 times of independent experiments.
Data exclusions	No data were excluded.
Replication	All attempts at replication were successful.
Randomization	All samples for experiment was allocated randomly.
Blinding	All samples (mouse and cultured cells) for experiment were blinding to group allocation.

Reporting for specific materials, systems and methods

We require information from authors about some types of materials, experimental systems and methods used in many studies. Here, indicate whether each material, system or method listed is relevant to your study. If you are not sure if a list item applies to your research, read the appropriate section before selecting a response.

Materials & experimental systems

n/a	Involved in the study	
☒	☐	Antibodies
☐	☒	Eukaryotic cell lines
☒	☐	Palaeontology and archaeology
☐	☒	Animals and other organisms
☒	☐	Clinical data
☒	☐	Dual use research of concern

Methods

n/a	Involved in the study	
☒	☐	ChIP-seq
☒	☐	Flow cytometry
☒	☐	MRI-based neuroimaging

Eukaryotic cell lines

Policy information about cell lines and Sex and Gender in Research

Cell line source(s)	LS174T is a cell exhibiting epithelial morphology that was isolated from the colon of a White, 58-year-old, female adenocarcinoma patient with colorectal cancer.
Authentication	All cell lines used in this study was authenticated.

Mycoplasma contamination	All cell lines used in this study was tested for mycoplasma contamination and showed negative.
Commonly misidentified lines (See ICLAC register)	MT-3 cell lines, Yu et al, 2015.

Animals and other research organisms

Policy information about studies involving animals; ARRIVE guidelines recommended for reporting animal research, and Sex and Gender in Research

Laboratory animals	Six-week-old C57BL6 male mice.
Wild animals	The study did not involve wild animals.
Reporting on sex	We have used only male mouse in this study. But we have preliminary tested both male and female mouse and showed same results.
Field-collected samples	The study did not involve samples collected from the field.
Ethics oversight	The animal experiments were approved by the Institutional Animal Care and Use Committee of Kyoto Prefectural University of Medicine (Kyoto, Japan) under Assurance Number M 2021-129.

Note that full information on the approval of the study protocol must also be provided in the manuscript.

This checklist template is licensed under a Creative Commons Attribution 4.0 International License, which permits use, sharing, adaptation, distribution and reproduction in any medium or format, as long as you give appropriate credit to the original author(s) and the source, provide a link to the Creative Commons license, and indicate if changes were made. The images or other third party material in this article are included in the article's Creative Commons license, unless indicated otherwise in a credit line to the material. If material is not included in the article's Creative Commons license and your intended use is not permitted by statutory regulation or exceeds the permitted use, you will need to obtain permission directly from the copyright holder. To view a copy of this license, visit http://creativecommons.org/licenses/by/4.0/
